# L’œdème aigue post chirurgical: complication redoutable

**DOI:** 10.11604/pamj.2015.20.229.5840

**Published:** 2015-03-12

**Authors:** Najib Bouhabba, Mustapha Bensghir, Salaheddine Fjouji, Hicham Azendour, Charki Haimeur

**Affiliations:** 1 Département d'Anesthésie Réanimation, Hôpital Militaire d'Instructions Mohamed V, Rabat, Maroc

**Keywords:** Œdème aigue du poumon post obstructif, l′extubation, reintubation, post osbtructive pulmonary edema, extubation, reintubation

## Abstract

L’œdème aigu pulmonaire (OAP) post-obstructif est une complication respiratoire grave qui doit être reconnue par les anesthésistes et les réanimateurs a fin de permettre une prise en charge diagnostique et thérapeutique précoce. Nous rapportons un cas survenu chez un jeune patient en postopératoire d'une appendicectomie. Immédiatement après l'extubation le patient a développé une détresse respiratoire, une désaturation et des signes auscultatoires et radiologiques évoquant un œdème aigu du poumon. Un bilan étiologique, notamment cardiaque, est revenu normal et l’évolution était favorable dans les heures qui suivent après reintubation et traitement par diurétiques. Le diagnostic de l'OAP post-obstructif était alors retenu. A travers ce cas et une revue de la littérature, les auteurs mettent en relief cette complication redoutable nécessitant une prise en charge précoce.

## Introduction

L'œdème aigue du poumon (OAP) post-obstructif est une complication rare survenant chez 0,1% des patients après l'extubation [1]. Le développement brutal de fortes pressions négatives intra-thoraciques secondaire a l'obstruction des voies aériennes supérieures entraine une extravasation liquidienne intra-pulmonaire [2]. Cet événement inattendu peut avoir des conséquences létales, sa prévention passe par une prise en charge diagnostique et thérapeutique précoce. Cette observation clinique rapporte un cas d OAP post-obstructif compliquant une chirurgie d appendicite aiguë.

## Patient et observation

Un homme de 37 ans, a été admis dans un tableau de syndrome appendiculaire, le bilan biologique fait d'une numération formule montrait une hyperleucocytose à prédominance de polynucléaires neutrophiles, et l’échographie abdominale un épaississement de la paroi appendiculaire. Cliniquement, le patient ne présentait aucun critère d'intubation difficile. Six heures après le patient a été admis au bloc opératoire, l'induction de l'anesthésie était faite par fentanyl: 250 microgramme (µg), thiopental (400 mg) et cisatracurium (10 mg), l'intubation trachéale était facile avec une sonde de 7,5 mm. L'entretien de l'anesthésie était réalisé par l'isoflurane dans un mélange O2/N2O (50%/50%). Une appendicectomie conventionnelle a duré 65 min sans aucun incidents per-opératoire. Les apports per opératoire étaient de 1000 ml de SS 0,9% l'analgésie postopératoire était assurée par le paracétamol 1g. Apres réveil complet et décurarisation le patient a été extubé en salle de surveillance post interventionnelle, immédiatement après l’éxtubation le patient a développé une détresse respiratoire avec une dyspnée inspiratoire sans stridor, tirage sus sternal et une désaturation à 70%. Le patient était tachycarde à 110 bpm (battement par minute), avec une tension artérielle stable 130/80 mm Hg. L'auscultation pleuro-pulmonaire avait trouvé des râles crépitants bilatérales et diffuses.

L'apport en oxygène a haut débit 15l/min par un masque a haute concentration a permis d'augmenter partiellement et temporairement la saturation, une nébulisation d'adrénaline a été également pratiquée. Une radiographie pulmonaire a été réalisé a la salle de surveillance post interventionnelle avait objectivé des infiltrats alvéolo-interstitielle bilatérales et diffuses en ailles de papillon évoquant un œdème aigu du poumon. Apres 30 min de l’éxtubation et devant la dégradation de l’état respiratoire, l'apparition d'une agitation témoignant d'une hypoxémie profonde et l'apparition de sueurs profuses évoquant une hypercapnie. Le patient a été ré-intubé avec issu de secrétions mousseuses et hémoptoiques par la sonde d'intubation et lors de l'aspiration trachéale initiale, la ventilation mécanique en mode volume contrôlé avec les paramètres suivants: VT: 450ml, FR: 15c/min et une PEEP: 10 mm hg avait engendrée une pressions de crête a 37mmhg et une pression du plateau a 29 mmhg. la fraction inspirée en oxygène (Fio2) a été réglée initialement a 70% pour avoir une saturation supérieure a 90%. Une dose de 80mg de furosémide a été administrée en intraveineux après un sondage urinaire puis le patient a été transféré en milieu de réanimation après sédation par le midazolam en pousse seringue électrique. L’électrocardiogramme réalisé était normal et l’échocardiographie trans-thoracique était sans particularité notamment pas d'anomalie valvulaire et une fonction systolique globale du ventricule gauche conservée. Le diagnostic d un OAP post-obstructif a été évoqué, deux doses de furosémide (40 mg) ont été données à 4 heures d'intervalle avec une restriction des apports par voie intraveineuse. Après 12 heures de ventilation mécanique l'examen clinique avait montré une disparition des râles crépitants avec une amélioration de l'oxygénation (Sp02: 98% sous Fio2 a 40%) la radiographie de contrôle avait objectivé un nettoyage radiologique. Apres arrêt de la sédation et un réveil complet le patient a été extubé et transféré au service de chirurgie après 24 heures de surveillance sans anomalie notable.

## Discussion

L'OAP post obstructif ou l'OAP à pression négative (OAPPN) est une entité pathologique qui a été décrite ces dernières années. Elle est due a une obstruction des VAS survenant dans la période postopératoire, le plus souvent dans les premières 15 minutes suivant l'extubation [1, 3, 4]. La fréquence de cette complication est estimée a 0,1% des patients ayant subit une anesthésie générale [1, 4, 5], cependant cette prévalence est sous estimée vu la méconnaissance de cette complication dans le contexte anesthésique [5] L'OAPPN survient le plus souvent chez le sujet jeune en bon état général, capable de développer de fortes pressions négatives intra-thoracique en réponse a une obstruction aigue des VAS [4, 6] Même si les causes de cette complication sont multiples, le laryngospasme reste la plus fréquente, il est responsable d environ 50% des cas rapportés dans la littérature [4, 5], la curarisation et l'anesthésie résiduelle sont aussi incriminées car ils peuvent induire une hypotonie des muscles des VAS, soit par obstruction des VAS via un corps étranger, ou par oubli de matériel prothétique. Certains types de chirurgies sont plus pourvoyeuses de cette complication et surtout la chirurgie de la face et la chirurgie du cou [4–6].

La physiopathologie d l OAPPN est mieux connu de nos jours, le ««primus movens»» est une obstruction aigue des VAS contrariée par des forces inspiratoires, entrainant une importante négativation des pressions intra-thoraciques et précisément intra-pleurales qui peut aller jusqu'à 100mmhg chez le sujet jeune, le résultat est une augmentation du retour veineux au cœur droit (précharge) avec majoration considérable de la pression veineuse intra-pulmonaire, un gradient hydrostatique se crée ainsi entre l interstitium et le lit capillaire pulmonaire conduisant a une extravasation liquidienne [7, 8]. L'hypoxie qui en résulte entraine une vasoconstriction périphérique par stimulation adrénergique avec augmentation de la post-charge majorant ainsi le gradient hydrostatique [9]. OAPPN se développe habituellement peu ou immédiatement après l'extubation, bien que certaines manifestations aient été signalées jusqu’à 1 heure après l'extubation. Cette manifestation tardive peut s'expliquer par une protection initiale liee à des efforts d'expiration vigoureuse créant une pression positive ("auto-PEEP") qui s'oppose a la transsudation liquidienne [9, 10]. Dans le cas de notre patient, les manifestations sont apparues immédiatement après l'extubation, même si aucun signe de stridor ou d'un traumatisme n’était évident lors de la manipulation des voies aériennes supérieures, le patient était jeune et sportive, et capable de générer des forces inspiratoires considérables aboutissant à des fortes pressions négatives pleurales menant à un œdème pulmonaire. Le traitement de L OAPPN comporte initialement une assistance ventilatoire, dans les cas graves l intubation avec ventilation en pression positive s impose a fin d'améliorer les échanges gazeux par le recrutement alvéolaire [2]. La ventilation non invasive reste une alternative dans les détresses respiratoires modérées. Le traitement médical reste encore controversé, les diurétiques à doses modérées sont fréquemment utilisés [2, 4, 6], les corticostéroïdes par voie générale et les B2 mimétiques inhalés sont également utilisés [4, 10]. Dans le cas de notre patient l’évolution était favorable sous ventilation à pression positive avec adjonction des diurétiques, la résolution de l OAPPN s'est faite en 12 heures environ.

## Conclusion

L OAP post-obstructif, est une complication anesthésique qui peut aggraver un acte chirurgical simple. Le diagnostic doit être toujours évoqué devant un tableau de détresse respiratoire post chirurgical. La prévention, la rapidité, et l'efficacité du traitement permettent d'assurer une prise en charge efficace, et curative du patient.
